# The doctor's dilemma: Truth telling

**DOI:** 10.4103/0019-5545.42392

**Published:** 2008

**Authors:** G. Swaminath

**Affiliations:** Department of Psychiatry, Dr. B. R. Ambedkar Medical College, Kadugondanahalli, Bangalore - 560 045, Karnataka, India

## THE TRUTH, THE WHOLE TRUTH, AND NOTHING BUT THAT?

Even as we glory in being in the age of right to information, I am occasionally reminded of a story I read during my school days, “The doctor's word” by Narayan.[1] It narrates the dilemma of a doctor who diagnoses his friend, who totally trusts him, to be critically ill. The doctor realizes that his friend has a good chance of recovery if he does not deteriorate over the night. The ethical dilemma relates to whether the doctor should inform the patient of his criticality and lessen hope or deliberately mislead him, and improve chances of survival by instilling hope. The patient has expressed his desire to write a will, should his survival be improbable. Thanks to the decision taken by the doctor to deceive, the patient “finds” hope and survives. The doctor fulfils his “Hippocratic Oath,” “reducing patient harm by not revealing upsetting conditions.”

Trust is belief that someone is honest and will not cause harm, and it is with this conviction that patients confide in doctors and seek advice.[2] A treatment is more likely to work (placebo effect) if there is faith in the doctor and the prescribed medication, hence trust is important for the patient's wellbeing. In the long running MORI annual poll,[3] the British public have voted for doctors as the most trusted professionals, as well as being the most likely to tell the truth. Despite this there is consistent evidence that patients do not always follow doctors' advice, and do not tell doctors when they are not doing so.[2] This reflects a “critical trust” where trust is rarely given unconditionally allowing the lingering of at least faint skepticism.

Generally, children are taught to tell the truth absolutely and to avoid lies. Doctors who condemn lying may sometimes misrepresent the patient's condition without actually telling a lie.[4] They may withhold information about the patient's condition or proposed interventions, or give partial information which is literally true, yet deceptive. This is more common with illness which has stigmatizing diagnosis or poor prognosis.

Access to truth is a right (because respect demands it), a utility (to enable making of informed judgments) and a kindness (as lies poison relationships, resulting in withdrawal from constructive liaisons).[5]

Truth could be sabotaged by different ways. For example, while advising a psychotic person about clozapine and its adverse effects, the doctor might take one of these positions with varying forms and degrees of untruthfulness:[4]

- Claim no dangerous side effects (lying).
- Emphasize the absence of extrapyramidal symptoms, talk about and suggest blood tests required but not clarify why, use technical jargon, omit important qualifying information, present statistics in a misleading way (deception).
- Warn vaguely of agranulocytosis, suggest blood tests claiming it is simple and routine, downgrade probabilities of risk or gravity, not clarify if patient has understood or not (misrepresentation).
- Offer no information on side effects till the patient explicitly asks (nondisclosure).

The withholding the truth by physicians is a form of medical paternalism and is adopted to protect the patient from physical/emotional harm. This paternalism assists generate an optimism which transcends the immediate crisis and promotes decision making. It seems that false optimism about recovery and the absence of side effects (in this case the drug) is the result of an association between doctors' activism and patients' adherence to the recovery plot.[6] This allows both to not acknowledge explicitly what they should know and can know,[6] and play out a show orchestrated by the doctor.

Does this mean that without the patient's consent, for paternalistic reasons truth can be shunted out? Some authors[5] justify this overriding of truth by a temporary more important value, i.e., recipient survival, community survival, and the ability to absorb the full impact of the truth at a particular time. This, however, is temporary and can be played under certain limited conditions, only because the respect for the person (and therefore an inalienable right of the person) is a fundamental value in all relationships.

## PRIMUM NON NOCERE

The goal of medicine is to receive/provide help for an illness in a way no further harm is done to the patient, especially in his vulnerable state. As vulnerability is also because of an unequal relationship between doctor and patient, the patient should be assisted back to a state of human equality, free from dependency by restoring the patient's autonomy. The healing relationship enters into a calculus of values wherein the respect for the right to truth of the patient is weighed against impairing the restoration of autonomy by the truth.[5]

In short-term relationships between doctors and patients, where decisions have to be made in a compressed period, there is less opportunity to worry about the impact of truth on the patient. However, in long-term relationships, such as those which psychiatrists develop with patients and their care givers, truth is likely to be withheld for compassionate reasons more readily. In this relationship, there is a stronger bond as the focus of management is on the illness rather than the disease. In this context, it is often more justifiable to withhold the truth temporarily in favor of more important long-term values, which are known in the relationship.[5] However, it is important to remember here that “truth will always out.”

Narayan's story cited at the beginning dramatically brings to focus the ethical dilemmas in truth telling, the outcome was favorable to the patient, hence, for the doctor as well. Today, few doctors would take such a risk. Fear of litigation is not the only reason for such reluctance. The other reason could be the improved technology which has reduced the powerlessness of the healer, who need not now treat full disclosure as equivalent to a death sentence.[5] But primarily over time, the autonomy of the patient has justifiably taken precedence over beneficence and nonmalfeasance. Many writers of contemporary bioethics believe that all intentional suppression of pertinent information violates a patient's autonomy rights and violates the fundamental duties of a physician.
